# Clinical Differences between Younger and Older Adults with HIV/AIDS Starting Antiretroviral Therapy in Uganda and Zimbabwe: A Secondary Analysis of the DART Trial

**DOI:** 10.1371/journal.pone.0076158

**Published:** 2013-10-02

**Authors:** Sujal M. Parikh, Ekwaro A. Obuku, Sarah A. Walker, Aggrey S. Semeere, Brandon J. Auerbach, James G. Hakim, Harriet Mayanja-Kizza, Peter N. Mugyenyi, Robert A. Salata, Cissy M. Kityo, on behalf of the DART Trial Team

**Affiliations:** 1 Joint Clinical Research, Centre, Kampala, Uganda; 2 Medical Research Council Clinical Trials Unit, London, United Kingdom; 3 University of Michigan Medical School, Ann Arbor, Michigan, United States of America; 4 Case Western Reserve University, Department of Medicine, Cleveland, Ohio, United States of America; 5 Makerere University College of Health Sciences, Department of Medicine, Kampala, Uganda; 6 Makerere University College of Health Sciences, Infectious Diseases Institute, Kampala, Uganda; 7 Harvard University, Harvard Medical School, Boston, Massachusetts, United States of America; 8 University of Zimbabwe Clinical Research Centre, College of Health Sciences, Harare, Zimbabwe; University of Pittsburgh, United States of America

## Abstract

**Objective:**

Clinical and immunological data about HIV in older adults from low and middle income countries is scarce. We aimed to describe differences between younger and older adults with HIV starting antiretroviral therapy in two low–income African countries.

**Methods:**

**Setting**: HIV clinics in Uganda and Zimbabwe. **Design**: Secondary exploratory cross-sectional analysis of the DART randomized controlled trial. **Outcome Measures**: Clinical and laboratory characteristics were compared between adults aged 18-49 years (younger) and ≥ 50 years (older), using two exploratory multivariable logistic regression models, one with HIV viral load (measured in a subset pre-ART) and one without.

**Results:**

A total of 3316 eligible participants enrolled in DART were available for analysis; 219 (7%) were ≥ 50 years and 1160 (35%) were male. Across the two adjusted regression models, older adults had significantly higher systolic blood pressure, lower creatinine clearance and were consistently less likely to be females compared to younger adults with HIV. Paradoxically, the models separately suggested that older adults had statistically significant (but not clinically important) higher CD4+ cell counts and higher plasma HIV–1 viral copies at initiation. Crude associations between older age and higher baseline hemoglobin, body mass index, diastolic blood pressure and lower WHO clinical stage were not sustained in the adjusted analysis.

**Conclusions:**

Our study found clinical and immunological differences between younger and older adults, in a cohort of Africans starting antiretroviral therapy. Further investigations should explore how these differences could be used to ensure equity in service delivery and affect outcomes of antiretroviral therapy.

## Introduction

Globally, the proportion of older adults is increasing [1]. In regard to HIV, persons 50 years and above are considered “older adults” [2–4]. In this fourth decade of the AIDS epidemic, the HIV–infected population is growing older primarily due to successful antiretroviral therapy (ART) but also among those newly diagnosed [5]. Reports from high-income countries show that adults over 50 years doubled from 10-15% of the HIV–infected population in 2005, to about 30% by 2010 [6,7]. This phenomenon has also been observed in sub–Saharan Africa [8].

Older adults with HIV present with different clinical, immunological and even social profiles which potentially influence outcomes [9–14]. For example, adults aging with HIV experience multiple co-morbidities with resultant poly-pharmacy, may respond differently to ART, and are commonly stigmatized or socially isolated. However, existing evidence on aging and HIV/AIDS is largely from high income countries. The few publications from sub–Saharan Africa do not provide as much information regarding some of the co-morbidities such as Hepatitis B and C co-infection, renal dysfunction, hypertension or viral loads [8,15–17]. In order to explore these issues of HIV patients initiating ART, we compared clinical and immunological differences between older (≥ 50 years) and younger (18-49 years) adults starting ART in Uganda and Zimbabwe which are low income countries in sub–Saharan Africa.

## Methods

### Study design, procedures and setting

In this secondary data analysis, we used information from the Development of Anti-Retroviral Therapy in Africa (DART) trial. DART was an open randomized trial comparing two management strategies for monitoring ART, and details of the methods have been reported elsewhere [18]. Briefly, 3316 eligible symptomatic (WHO stage 2–4) HIV–infected adults (≥18 years) with CD4+ counts less than 200 cells/ml who reported no previous ART apart from to prevent mother–to–child transmission, were enrolled from three centres in Uganda and one centre in Zimbabwe from 15 January 2003 to 28 October 2004. Viral loads (n=968 at baseline) were done retrospectively using stored samples, and were not used for clinical management.

### Statistical analysis

We conducted this exploratory analysis to identify variables that differed significantly between the younger and older adults with HIV (18-49 versus >50 years) at enrollment into ART care. We summarized clinical measures (sex, systolic and diastolic blood pressure, body mass index, WHO clinical stage), laboratory results (hemoglobin, CD4+ cell counts, viral load, creatinine, alanine and aspartate amino–transferase), infectious (hepatitis B surface antigen and anti-hepatitis C) and non-communicable (hypertension, overweight and obesity) co-morbidities using frequencies, percentages and measures of central tendency as appropriate. We referred to World Health Organization cut offs for hypertension, overweight and obesity. We computed creatinine clearance using the Cockcroft–Gault equation [19]. We then explored associations between age (18-49 versus >50 years) and each variable using logistic regression. Tests were two–sided and considered significant if P<0.05. Variables with P<0.2 in univariate analysis were included in a multivariable logistic regression model to estimate the adjusted Odds Ratios (aOR) and their 95% confidence intervals (95% CI). We excluded creatinine and body mass index (BMI) from adjusted analyses due to their significant correlation with creatinine clearance (r^2^ =-0.6 and r^2^=0.33, respectively, both p<0.0001). Because not all participants had HIV-1 RNA measures taken, we employed two adjusted regression models. Model 1 incorporated all variables eligible for inclusion from the univariate analysis (P<0.2), except viral load, creatinine and BMI (as above). An interaction term between sex and hemoglobin was included since a previous analysis showed that women had significantly lower hemoglobin in DART [20]. Model 2 included viral load measurements into model 1 in the subgroup of participants with pre-ART HIV-1 RNA levels available.

### Ethical considerations

The DART trial was approved by ethics committees in Uganda, Zimbabwe and United Kingdom. This secondary analysis was approved by the Joint Clinical Research Centre Institutional Review Board in Uganda. Written informed consent was obtained from each enrolled participant for trial participation and provision and analysis of data.

## Results

### Baseline characteristics

3316 eligible participants were included in the DART trial. Overall, the majority were females (65%) and the mean age (SD) was 37.6 years (7.8) with 219 (7.0%) of the participants aged ≥ 50 years or older. Only 968 (29.2%) participants had baseline viral loads measured and the mean (SD) was 5.4 (0.7) log 10 copies/mL. Further details are in table 1.

**Table 1:** Characteristics at ART initiation stratified by age < 50 years and age ≥ 50 years.

**Variable**	**N = 3316**	**18-50 years**	**≥ 50 years**	**P-Value**
n (%)		3097 (93)	219 (7)	
Age (years, mean, SD)	37.6 (7.8)	36.3 (6.3)	55.1 (4.4)	P<0.001
Sex				
Male (%)	1160 (3,5)	1054 (90.9)	106 (9.1)	P<0.001
Female (%)	2156 (65)	2043 (94.8)	113 (5.2)	
BMI				
mean (SD)	21.7 (3.8)	21.6 (3.8)	22.5 (3.9)	P<0.001
Blood Pressure (mm Hg)				
Systolic, mean (SD)	110.6 (14.7)	109.9 (13.9)	120.9 (20.2)	P<0.001
Diastolic, mean (SD)	72.3 (11.1)	71.9 (10.8)	77.9 (13.6)	P<0.001
WHO Clinical Stage (%)				
II	673 (20)	620 (92)	53 (8)	P=0.077
III	1864 (56)	1737 (93)	127 (7)	
IV	779 (24)	740 (95)	39 (5)	
CD4+ (cells/µL)				
median (IQR)	86 (81–139)	85 (30–138)	92 (45–147)	P=0.222
Viral Load (log_10_ cp/mL)				
mean (SD)	5.4 (0.7)	5.4 (0.7)	5.7 (0.5)	P=0.003
Hemoglobin (g/dL)				
mean (SD)	11.5 (1.7)	11.5 (1.8)	11.7 (1.5)	P=0.078
Creatinine (mg/dL)				
mean (SD)	0.91 (0.27)	0.90 (0.27)	0.98 (0.26)	P<0.001
ALT (mmol/L)				
median (IQR)	25 (18–36)	25 (18–36)	23 (17–33)	P=0.008
AST (mmol/L)				
median (IQR)	34 (26–48)	34 (26–48)	34 (25–44)	P=0.895
Hepatitis B virus				
pos (%)	302 (9.3)	290 (96)	12 (4)	P=0.053
neg (%)	2,950 (90.7)	2,747 (93.1)	203 (6.9)	
Hepatitis C virus				
pos (%)	77 (2.4)	69 (89.6)	8 (10.4)	P=0.177
neg (%)	3,176 (97.6)	2,969 (93.5)	207 (6.5)	

Abbreviations: ALT – Alanine transaminase; AST – Aspartate transaminase; BMI – Body Mass Index; CD4+ – Cluster of Differentiation; CI – Confidence Interval; cp – copies; IQR – Inter Quartile Range; neg – negative; OR – Odds Ratio; pos – positive; SD – Standard Deviation; WHO – World Health Organization; *d*L – deciliter; g – grams; Hg – mercury; mmol – millimols; mL − milliliter; mm – millimeters; µL – microliter

* *n=968 (< 50 years n = 912; > 50 years n = 56*)*.*

### Prevalence of co-morbidities

The prevalence of systolic and diastolic hypertension was 21.3% and 19.0% for older adults; and 9.2% and 3.5% for younger adults with HIV (both, p<0.001) respectively. 24.0% of older adults and 14.8% younger adults were either overweight or obese (p<0.001). The prevalence of anti-hepatitis C virus sero-positivity was 3.7% and 2.3% for older and younger adults respectively (p=0.17); whilst that for hepatitis B surface antigen was 5.6% and 9.6% respectively (p=0.05). Finally, the proportion of having at least two co-morbidities (communicable and or non-communicable) was 11.4% and 5.1% among older and younger adults respectively (p<0.001) (Data not tabulated).

### Differences between older (≥50 years) and younger (18-49 years) adults with HIV and AIDS

In the adjusted analysis without viral load data (table 2, model 1), there were significantly fewer females among older adults (aOR: 0.03, 95% CI: 0.00-0.19, p<0.001). Older adults had significantly higher systolic blood pressure (aOR: 1.05, 95% CI: 1.03-1.06, p<0.001), higher CD4+ cell counts (aOR: 1.00, 95% CI: 1.00-1.01, p=0.009) and lower creatinine clearance (OR: 0.97, 95% CI: 0.96-0.97, p<0.001). The association between higher hemoglobin and older age remained significant among females, sustaining the effect modification even after adjustment (heterogeneity p=0.003).

**Table 2:** Logistic regression model for characteristics at ART initiation for adults who were older (≥ 50 years) compared to younger ones (18-50 years) (N=3316).

**Variable**	**Unadjusted**			**^§^Adjusted Model 1 (n=3184)**			Ω **Adjusted Model 2** (**n=943**)		
	**OR**	**95% CI**	**p-value**	**aOR**	**95% CI**	**p-value**	**aOR**	**95% CI**	**p-value**
Female	0.55	0.42-0.72	**<0.001**	0.03	0.00-0.19	**<0.001**	0.01	0.00-0.38	**0.016**
BMI (Kg/M^2^)	1.06	1.02-1.09	**0.001**	–	–	–	–	–	–
Syst. BP (mm Hg)	1.04	1.03-1.05	**<0.001**	1.05	1.03-1.06	**<0.001**	1.06	1.03-1.09	**<0.001**
Diast. BP (mm Hg)	1.04	1.03-1.06	**<0.001**	0.99	0.98-1.02	0.728	1.00	0.96-1.03	0.597
α WHO stage	0.79	0.64-0.97	**0.027**	0.98	0.78-1.24	0.878	1.19	0.56-2.56	0.649
CD4+ (cells/µL)	1.00	1.00-1.01	**0.009**	1.00	1.00-1.01	**0.034**	1.00	1.00-1.01	0.359
*VL (log 10 cp/mL)	1.97	1.27-3.05	**0.002**	–	–	–	2.51	1.07-5.87	**0.034**
Hb (g/dL)	1.07	0.99-1.16	0.078	0.91	0.82-1.03	0.125	0.78	0.60-1.00	0.108
Female Hb (g/dL)	1.28	1.09-1.51	**0.003**	1.29	1.09-1.54	**0.003**	1.51	1.06-2.18	**0.024**
Cr (mg/dL).	2.19	1.48 - 1.26	**<0.001**	–	–	–	–	–	–
Cr. clear (mL/min).	0.97	0.96-0.98	**<0.001**	0.97	0.96-0.97	**<0.001**	0.95	0.94-0.97	**<0.001**
ALT (mmol/L)	0.99	0.99-1.00	0.349	–	–	–	–	–	–
AST (mmol/L)	0.99	0.99-1.00	0.243	–	–	–	–	–	–
Hep. B sAg (pos)	0.56	0.31-1.01	0.056	0.60	0.32-1.11	0.101	0.28	0.04-2.20	0.225
Hep. C (pos)	1.66	0.79-3.50	0.181	1.52	0.67-3.36	0.302	1.54	0.25-6.86	0.602

Abbreviations: ALT – Alanine transaminase; AST – Aspartate transaminase; BMI – Body Mass Index; clear. – clearance; CD4+ – Cluster of Differentiation; CI – Confidence Interval; cp – copies; Cr. – Creatinine; IQR – Inter Quartile Range; Kg – Kilogramme; M^2^ – Meter; SD – Standard Deviation; WHO – World Health Organization; *d*L – deciliter; g – grams; Hg – mercury; mmol – millimols; mL − milliliter; mm – millimeters; µL – microliter * n=968 ( < 50 years n = 912; > 50 years n = 56) in log 10 copies per milliliter. § *Baseline adjusted model;* Ω *- model with viral load, interaction between gender and hemoglobin; and creatinine clearance (BMI excluded*) α − WHO stages 2, 3 & 4 included as a linear trend

Using viral load in the sub-population where this was available (table 2, model 2) the association between older age and higher systolic blood pressure and lower creatinine clearance was maintained (p<0.001). Additionally, females were significantly fewer in the older age group (p=0.025). The effect modification of sex in the association between older age and higher hemoglobin among females also remained significant (heterogeneity p=0.049). However, higher viral load (p=0.005) remained independently associated with older adults infected with HIV instead of CD4+, which no longer provided independent information (p=0.4).

## Discussion

HIV and aging is an inevitable global health challenge today [21]. The world population is aging to the extent that chronic diseases are on the rise and stretching health care budgets, particularly in sub–Saharan Africa [22]. Still, in low income countries like Uganda and Zimbabwe, the existing high burden of infectious diseases including HIV/AIDS, tuberculosis and malaria, presents a dual challenge to the health systems [22]. Therefore, innovative strategies to integrate care for older adults are urgently needed and understanding the characteristics of such a population is a sound starting point [3,23].

Our study explored clinical and immunological differences associated with older adults with HIV and AIDS starting antiretroviral therapy. Whilst such data are largely from the high income settings, few studies from sub–Saharan Africa have reported laboratory biomarkers for adults with HIV and AIDS older than 50 years. In the DART cohort, older adults had significantly higher systolic blood pressure, lower creatinine clearance, higher CD4+ cell counts, and higher plasma HIV–1 RNA viral copies/mL, compared to younger adults in the multivariable regression models. Women were also proportionately less well represented, despite making up the majority of the trial population.

That older adult females were significantly fewer in Zimbabwe and Uganda raises concern of gender inequity in accessing HIV and AIDS care in low income countries. This finding is corroborated in a recent study by Negin and colleagues who reported that older adults in Malawi were more likely to be male [24]. Indeed, older adult females with HIV are probably widowed, neglected and lack resources or the social fabric to reach HIV care and treatment services [25]. Alternatively, this could be a cohort effect depicting earlier mortality among females with HIV and AIDS compared to males such that, only those who survived accessed care. However, given results from a recent systematic review that men have worse outcomes even on ART, and that majority of trial participants were women, this is quite unlikely [26]. Noteworthy, this could be a consequence of earlier sexual debut associated with riskier sexual behavior and probably HIV infection in females compared to males [27,28]. On the contrary, older men may be more sexually active and with multiple partners, thus increasing their risk of HIV than older women [29]. Possibly, the DART recruitment procedures and avenues could have systematically excluded older women. Regardless of the underlying reason, our findings highlight the importance of HIV treatment and care interventions in Africa mainstreaming gender issues to improve access, even for females older than 50 years who are naturally no longer reproductive. Notably, menopause leads to changes in immune response, vaginal or cervical epithelium and secretions that may influence HIV transmission or progression among those who remain sexually active [30–32]. Hence, such programmes could consider expanding their maternal and child health paradigm to include women’s health. In relation to this, older adult females had consistently higher hemoglobin levels compared to the younger reproductive age group (potentially due to child-bearing in the latter) which underscores the point that their clinical needs are indeed different.

It is certainly not surprising that older adults had higher systolic blood pressure and lower creatinine clearance. Older age is characterized by general decline in organ (renal) function, narrowing and hardening of blood vessels [33–35]. However, recent evidence from Europe and America suggests that there is an excess risk of morbidity and mortality due to cardiovascular and renal diseases among HIV–infected compared to the non–HIV infected population [36,37]. Thus, older adults infected with HIV may require even closer monitoring of their renal and cardiovascular function, bearing in mind resultant poly-pharmacy and excess burden to the organs in the event of co-administration of treatment.

Although older adults had statistically higher CD4+ cell counts and higher plasma HIV–1 RNA levels compared to younger adults, clinically this should be interpreted cautiously. Coupled with the finding of fewer older adults in the lower WHO stages 3 and 4 univariate analysis, the higher CD4 counts suggests either earlier presentation and access to HIV care by older adults, or that more older adults died before they could access care, leaving those survivors who joined the DART trial with better underlying prognosis. The higher viral loads in older persons would tend to support the latter explanation, although they could also reflect decreased ability of the older immune system to respond to HIV [38].

This study had limitations, which can be addressed by future research. Relatively few older adults were recruited in the DART trial meaning power to detect differences between older and younger adults was low. In addition viral load results were available for only a third of the participants, thus limiting power of the findings in the second model. Certain data were not collected including substance abuse, mental health as well as specific biomarkers such as cholesterol, D–dimer, cystatin C and IL–6 all of which have been found to predict HIV treatment outcomes among older adults in western cohorts [39,40]. Lastly, a control group of non–HIV infected individuals would have provided vital information on the effect of HIV infection itself. Nonetheless, this is among the first studies on HIV and aging to explore clinical and immunological differences in a sub–Saharan African cohort.

## Conclusions

Our study explored and identified some bio-markers which may be useful in optimizing monitoring of care for older adults with HIV and AIDS. We also exposed information gaps for future research in cohorts of older adult Africans with HIV. These include establishing non–HIV infected cohorts to compare whether indeed in this setting, there is an interaction between aging and HIV in the clinical, immunological differences identified in our study; and which of these are optimal for reliably predicting outcomes of HIV treatment.
